# Supplementary material for: Causal effects of breast cancer risk factors across hormone receptor breast cancer subtypes: A two-sample Mendelian randomization study
Source: Cancer Epidemiol Biomarkers Prev. Author manuscript; Available in PMC 2025 Aug 14. (PMC12130805; doi:10.1158/1055-9965.EPI-24-1440)
Supplement: Supplementary data [file EMS207583-supplement-Supplementary_data.zip › epi-24-1440_supplemental_table_2_suppst2.docx]

Supplemental Table 2. Summary of Mendelian randomization studies on breast cancer subtype-specific risk factors

| *References* | *Risk Factors* | *Methods* | *Sample Size* | *Key Insights* |
| --- | --- | --- | --- | --- |
| *Chen, F. et al. (2022)* | 23 known and suspected risk factors including reproductive factors (age at menarche, age at menopause), body weight and anthropometrics (BMI, WHRadj BMI, height), lifestyle factors (physical activity, smoking, sleep duration, chronotype), and blood biomarkers (HDL-C, SHBG, fasting insulin, IGF-1, leukocyte telomere length, estrogens) | Two-sample MR using IVW, MR-PRESSO, MR-Egger regression | 86,627 BC patients; 59,378 ER+; 13,692 ER-; BCAC data includes 7,054 breast cancer-specific deaths. | **- Key Findings:**  Elevated risk factors include older age at menopause, younger age at menarche, higher height, smoking.  Protective factors include higher BMI, WHR, physical activity, morning preference chronotype.  **- Heterogeneity:** HDL-C and SHBG showed significant heterogeneity across subtypes. Age at menopause differed between luminal A and TNBC. Subtype-specific associations for fasting insulin and alcohol consumption, primarily with HER2-enriched cancers.  **- Subtype-Specific Findings:** HDL-C increased risk for luminal A, HER2-enriched, and TNBC. SHBG protective for luminal A-like but increased TNBC risk. |
| *Hayes, B. L. et al. (2022)* | Chronotype, bioavailable testosterone (BT), total testosterone (TT) | Univariable, bidirectional, and multivariable MR analysis | 133,384 BC cases, 113,789 controls; 244,207 females, 205,527 males for GWAS | **- Key Findings:** Morning-preference chronotype reduces breast and prostate cancer risk. BT and TT increase risk; bidirectional effects between chronotype and testosterone in females.  **- Heterogeneity:** No specific ER+/heterogeneity analyzed; focused on general associations with sex hormones.  **- Subtype-Specific Findings:** No specific subtype breakdown; focused on ER+/general risk. |
| *Shu, X. et al. (2022)* | 1890 circulating proteins, including ICAM1, PLA2R1, TXNDC12 | MR analysis using IVW method; protein quantitative trait loci (pQTL) data | 106,278 invasive cases, 91,477 controls (BCAC data); 3301 healthy individuals for proteins | **- Key Findings:** 98 circulating proteins associated with increased risk of breast cancer subtypes.  **- Heterogeneity:** Significant subtype-specific protein associations; heterogeneity in protein effects across subtypes.  **- Subtype-Specific Proteins:** 51 for luminal A/B; 14 for luminal B/HER2-negative; 11 for TNBC; 2 for HER2-enriched. |
| *Nounu, A. at al. (2022)* | Sex hormones (TT, BT, estradiol, SHBG, DHEAS, cortisol, progesterone, androstenedione) | Two-sample MR using IVW, MR-PRESSO, MR-Egger regression | 230,454 for TT GWAS, 189,473 for SHBG GWAS | **- Key Findings:** TT, BT, estradiol, and DHEAS increase risk of overall breast cancer (ER+ and luminal A-like subtypes).  **- Heterogeneity:** No strong pleiotropy; heterogeneity in TT, BT, and estradiol across ER+ and luminal subtypes; cortisol inversely associated with HER2-enriched.  **- Subtype-Specific Effects:** TT and BT protective for BRCA1-mutated TNBC; cortisol inversely linked to HER2-enriched. |
| *Bouras, E. et al. (2022)* | Cytokines: MCP1, IL-5, IL-7, IL-10, IL-16, RANTES, etc | Two-sample MR using GWAS meta-analyses for cytokines and cancers | 31,112 individuals of European descent for cytokines | **- Key Findings:** MCP1 increases overall breast cancer risk; IL-5, IL-7, IL-16 linked to HER2-positive; IL-10 linked to HER2-negative.  **- Heterogeneity:** No evidence of heterogeneity between cytokines and breast cancer subtypes. |
| *Fu, Y. et al. (2021)* | Circulating vitamin C concentration | Two-sample bi-directional MR with sensitivity analysis | 52,018 for vitamin C GWAS, up to 870,984 for cancer GWAS | **- Key Findings:** No causal association between circulating vitamin C and any examined cancer.  **- Heterogeneity:** No heterogeneity analyzed for vitamin C. |
