# Supplementary material for: Causal effects of breast cancer risk factors across hormone receptor breast cancer subtypes: A two-sample Mendelian randomization study
Source: Cancer Epidemiol Biomarkers Prev. Author manuscript; Available in PMC 2025 Aug 14. (PMC12130805; doi:10.1158/1055-9965.EPI-24-1440)
Supplement: Supplementary data [file EMS207583-supplement-Supplementary_data.zip › epi-24-1440_supplemental_table_1_suppst1.docx]

Supplemental Table 1. Summary of case-control and cohort studies on breast cancer subtype-specific risk factors

| *References* | *Risk Factors* | *Methods* | *Sample Size* | *Key Findings* |
| --- | --- | --- | --- | --- |
| *Jung, A. Y. et al. (2022)* | Reproductive factors (parity, age at first birth, breastfeeding, age at menarche/menopause) | Pooled analysis of 31 case-control/cohort studies using polytomous logistic regression | BCAC 23,353 cases, 71,072 controls | **- Age at menarche:** Younger menarche: higher risk of all subtypes.  **- Age at menopause:** Older menopause: higher luminal A-like risk, not TNBC. |
| *Gaudet, M. M. et al. (2018)* | Reproductive (lactation, age at menarche/menopause) and other (BMI) risk factors | Pooled analysis of 9 cohorts using multivariable, joint Cox proportional hazards regression models | 606,025 women, 11,741 invasive cases | **- BMI:** weight gain is more strongly linked to the risk of developing luminal B  **- Age at menarche/menopause:** Most strongly associated with increased risk of luminal A subtype. |
| *Anderson, K. N. et al. (2014)* | Reproductive risk factors (lactation, nulliparity, age at menarche/menopause, age at first birth, and breastfeeding) | Literature review | Ranged from cases: 374/controls: 2,533 to cases: 4,013/controls: 4,069 across individual studies | **- Age at menopause:** Older menopause: positively associated with hormone receptor positive tumors. |
| *McCarthy, A. M. et al. (2021)* | Established risk factors (race, reproductive history, prior breast problems, family history, breast density, and BMI) | Analysis of mammography screening population using Cox proportional hazard models | 198,278 women; 4,002 invasive cases | **- BMI:** Linked to increased risk of ER-/PR-/HER2- and HER2+ subtypes postmenopause.  **- Breast Density:** Associated with increased risk of all subtypes |
| *Schoemaker, M. J. et al. (2018)* | BMI | Pooled analysis of 19 prospective cohorts using Cox proportional hazards regression analysis | 758,592 premenopausal women; 13,082 cases | **- BMI:** Inverse linear association between BMI and premenopausal breast cancer risk.  - Stronger effect for ER+/PR+ tumors. |
| *Papadimitriou, N. et al. (2021)* | Diet (alcohol consumption, dairy products, milk, calcium, coffee) | Umbrella review of meta-analyses | Ranged from cases: 56/controls: 200,860 to cases: 117,514/controls: 5,197,851 across individual studies | **- Alcohol Consumption:** Positively associated with postmenopausal breast cancer. |
| *Gago-Dominguez, M. et al. (2016)* | Alcohol intake | Case-control study | 1,766 cases, 833 controls | **- Alcohol Consumption:** Increased risk of all subtypes, except for HER2 overexpressing.  - Stronger associations for lobular vs. ductal cancers. |
