# Supplementary material for: Causal effects of breast cancer risk factors across hormone receptor breast cancer subtypes: A two-sample Mendelian randomization study
Source: Cancer Epidemiol Biomarkers Prev. Author manuscript; Available in PMC 2025 Aug 14. (PMC12130805; doi:10.1158/1055-9965.EPI-24-1440)
Supplement: Supplementary data [file EMS207583-supplement-Supplementary_data.zip › epi-24-1440_supplemental_figure_7_suppsf7.pdf]

Age at menopause (years)

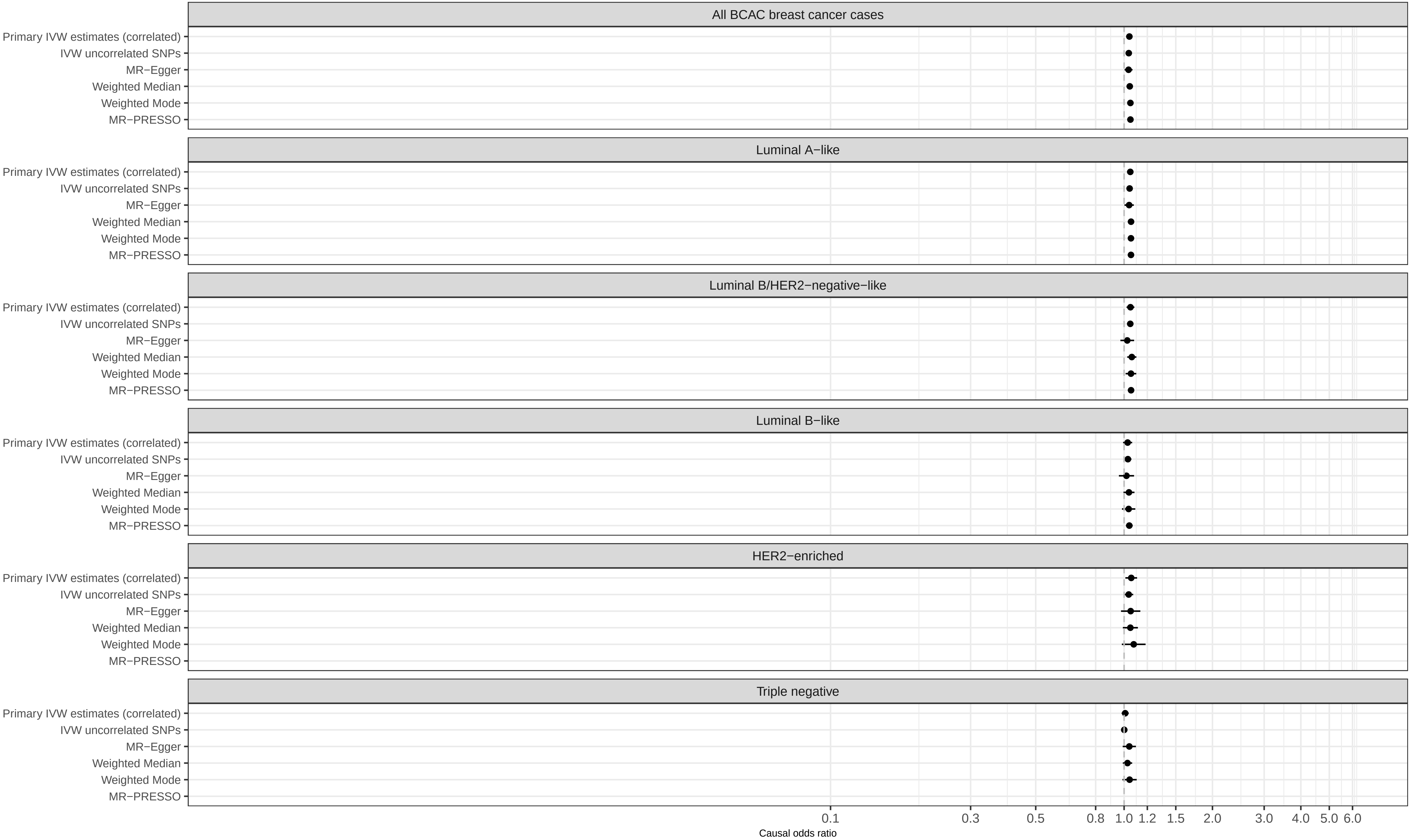

Supplemental Figure 7. Forest plots with causal effects of age at menopause on each hormone receptor breast cancer subtype across primary and secondary MR methods. Presented ORs and 95% CIs were calculated using the IVW method for correlated and uncorrelated SNPs, MR−Egger, Weighted Median, Weighted Mode, and MR−PRESSO. ORs for age at menopause correspond to a 1 year increase. The grey vertical dotted line indicates an OR of 1.00 (i.e., absence of a causal association).
