# Supplementary material for: Causal effects of breast cancer risk factors across hormone receptor breast cancer subtypes: A two-sample Mendelian randomization study
Source: Cancer Epidemiol Biomarkers Prev. Author manuscript; Available in PMC 2025 Aug 14. (PMC12130805; doi:10.1158/1055-9965.EPI-24-1440)
Supplement: Supplementary data [file EMS207583-supplement-Supplementary_data.zip › epi-24-1440_supplemental_figure_6_suppsf6.pdf]

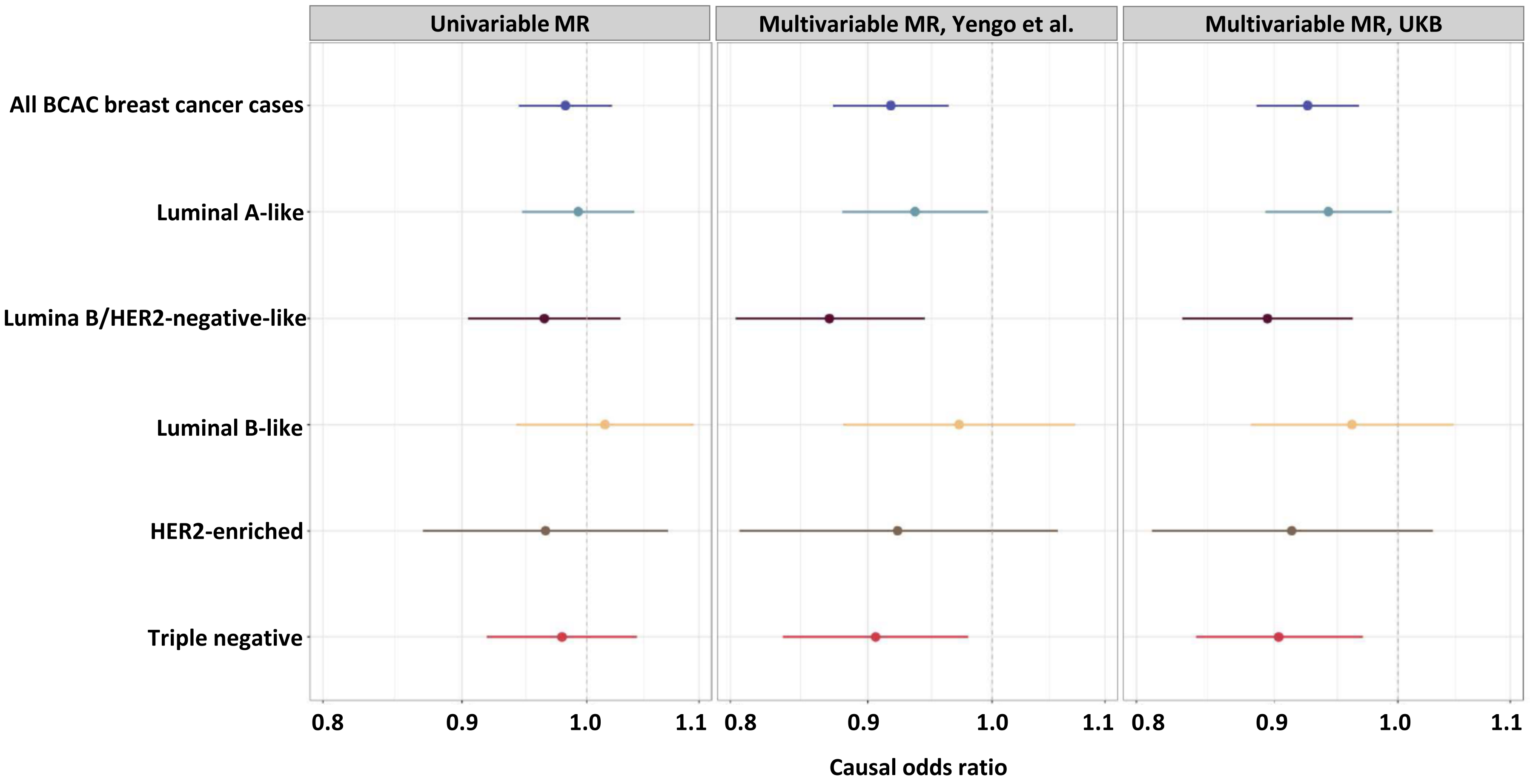

Supplemental Figure 6. Causal effect estimates for age at menarche from univariable and multivariable MR analyses. Forest plots show causal effect estimates (odds ratios) for age at menarche and overall breast cancer risk and hormone receptor subtypes calculated using univariable and multivariable MR analyses for body mass index (BMI). Two separate multivariable MR analyses were performed, which included different data sources for the BMI summary-level data. The middle panel show results from the multivariable MR using data from Yengo et al. (27), the right panel shows results from the analysis using UK Biobank data from Elsworth B. (https://gwas.mrcieu.ac.uk/datasets/ukb-b-19953/). Causal odds ratios and 95% CI are displayed across methods.
