# Supplementary material for: Causal effects of breast cancer risk factors across hormone receptor breast cancer subtypes: A two-sample Mendelian randomization study
Source: Cancer Epidemiol Biomarkers Prev. Author manuscript; Available in PMC 2025 Aug 14. (PMC12130805; doi:10.1158/1055-9965.EPI-24-1440)
Supplement: Supplementary data [file EMS207583-supplement-Supplementary_data.zip › epi-24-1440_supplemental_figure_5_suppsf5.pdf]

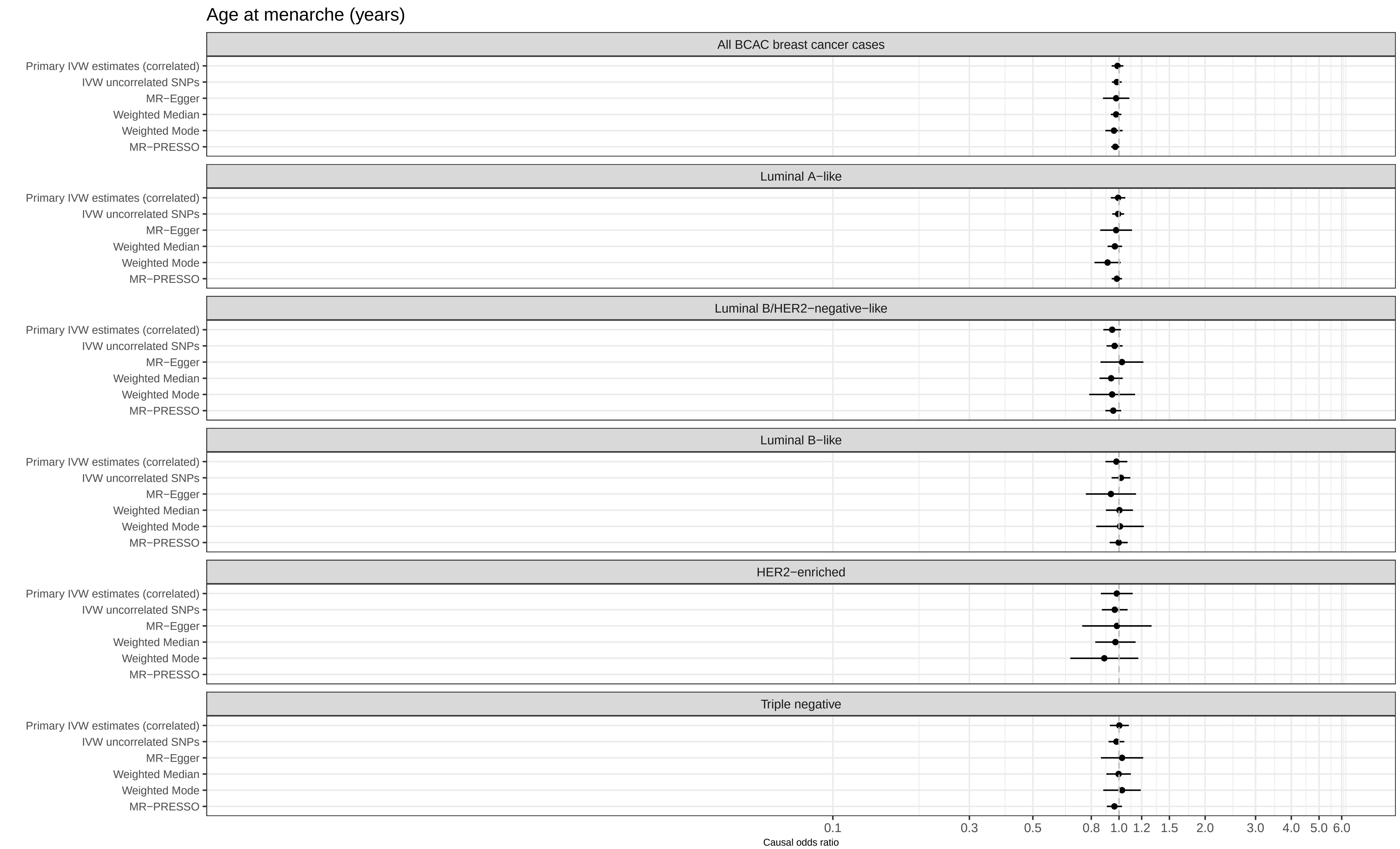

Supplemental Figure 5. Forest plots with causal effects of age at menarche on each hormone receptor breast cancer subtype across primary and secondary MR methods. Presented ORs and 95% CIs were calculated using the IVW method for correlated and uncorrelated SNPs, MR−Egger, Weighted Median, Weighted Mode, and MR−PRESSO. ORs for age at menarche correspond to a 1 year increase. The grey vertical dotted line indicates an OR of 1.00 (i.e., absence of a causal association).
