# Supplementary material for: Causal effects of breast cancer risk factors across hormone receptor breast cancer subtypes: A two-sample Mendelian randomization study
Source: Cancer Epidemiol Biomarkers Prev. Author manuscript; Available in PMC 2025 Aug 14. (PMC12130805; doi:10.1158/1055-9965.EPI-24-1440)
Supplement: Supplementary data [file EMS207583-supplement-Supplementary_data.zip › epi-24-1440_supplemental_figure_10_suppsf10.pdf]

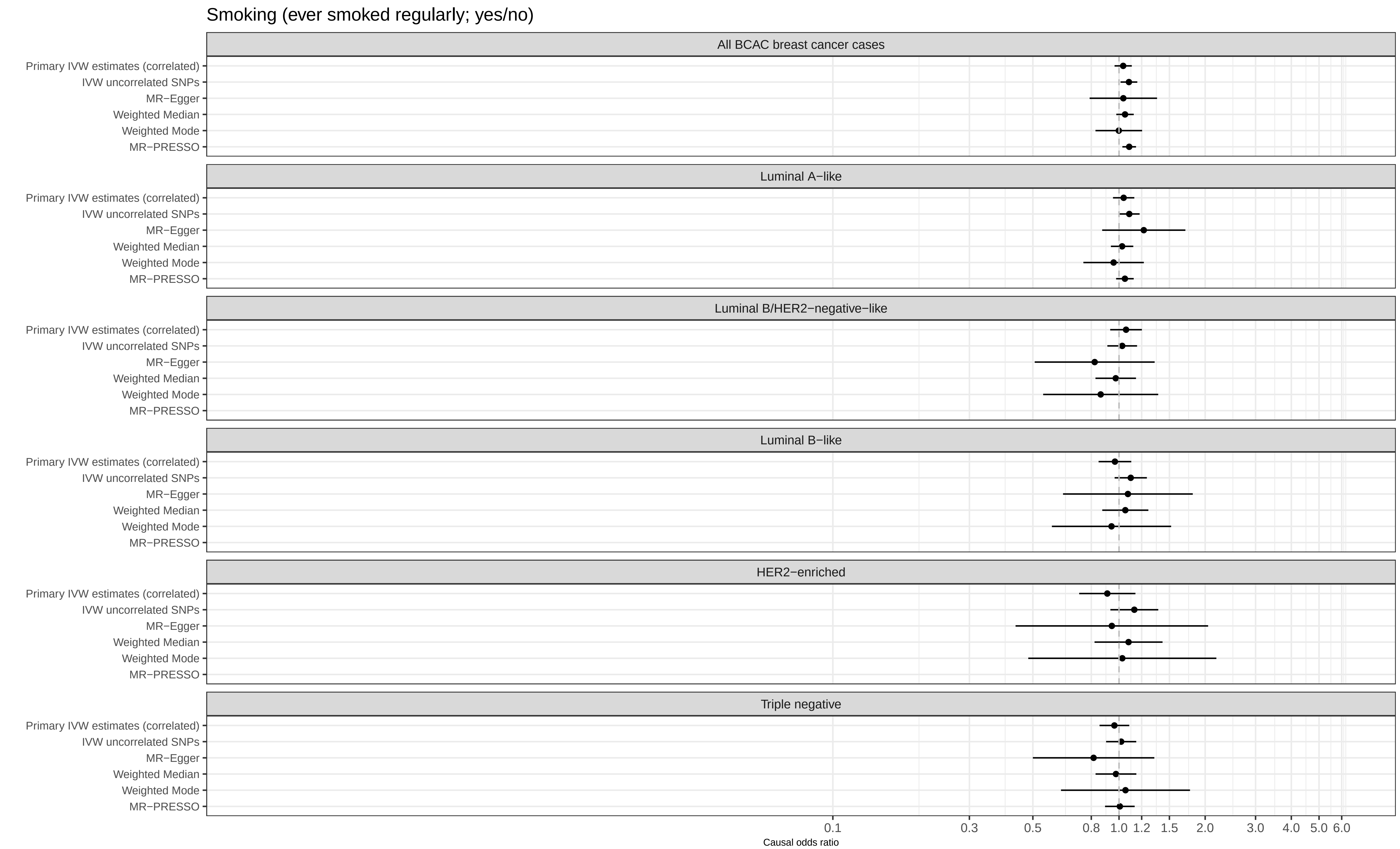

Supplemental Figure 10. Forest plots with causal effects of smoking on each hormone receptor breast cancer subtype across primary and secondary MR methods.
Presented ORs and 95% CIs were calculated using the IVW method for correlated and uncorrelated SNPs, MR−Egger, Weighted Median, Weighted Mode, and MR−PRESSO.
ORs for smoking correspond to a unit increase in the log odds. The grey vertical dotted line indicates an OR of 1.00 (i.e., absence of a causal association).
