# Supplementary material for: Causal effects of breast cancer risk factors across hormone receptor breast cancer subtypes: A two-sample Mendelian randomization study
Source: Cancer Epidemiol Biomarkers Prev. Author manuscript; Available in PMC 2025 Aug 14. (PMC12130805; doi:10.1158/1055-9965.EPI-24-1440)
Supplement: Supplementary data [file EMS207583-supplement-Supplementary_data.zip › epi-24-1440_supplemental_figure_1_suppsf1.pdf]

Inclusion criteria for genetic instruments	Breast cancer risk factors								
	Height	BMI	Type 2 diabetes	Age at menarche	Age at menopause	Percent breast density	Alcohol consumption	Smoking behaviour	Physical activity
Genome-wide signficiant in selected GWAS	N = 3,290	N = 941	N = 425	N = 389	N = 290	N = 20	N = 99	N = 378	N = 5
Available in BCAC summary statistics*	N = 3,146	N = 918	N = 404	N = 367	N = 257	N = 18	N = 96	N = 371	N = 5
SNPs retained after data harmonization**	N = 3,072	N = 892	N = 392	N = 355	N = 248	N = 18	N = 93	N = 353	N = 5
SNPs retained after calculation LD matrix***	N = 3,054	N = 884	N = 390	N = 322	N = 218	N = 16	N = 93	N = 349	N = 5
**Genetic instruments included in primary analyses (including correlated SNPs & LD matrices)**	N = 3,054	N = 884	N = 390	N = 322	N = 218	N = 16	N = 93	N = 349	N = 5
Genetic instruments included in secondary analyses (uncorrelated SNPs)	N = 1,304	N = 607	N = 360	N = 280	N = 193	N = 12	N = 80	N = 270	N = 5

Supplemental Figure 1. Overview of selection genetic instrumental variables. This figure presents the stepwise selection process for the genetic instruments that were used in this breast cancer (subtype specific) Mendelian randomization analyses. It displays the number of variants (N) retained at each step, across traits, from initial selection from genome-wide association studies (GWAS) until inclusion in our primary and secondary analyses.

*At this step genetic instrumental variables were excluded if they were not directly available in the BCAC GWAS summary statistics and if no suitable proxy SNP (r2 >= 0.8) was available for BCAC

** At this step genetic instrumental variables were excluded if they were palindromic SNPs (A/T or C/G alleles) with interemediate allele frequencies (> 0.4 and < 0.6)

***At this step genetic instrumental variables were excluded if they were not available on the 1000G reference panel
